# Very Long-Chain C24:1 Ceramide Is Increased in Serum Extracellular Vesicles with Aging and Can Induce Senescence in Bone-Derived Mesenchymal Stem Cells

**DOI:** 10.3390/cells8010037

**Published:** 2019-01-10

**Authors:** Andrew Khayrullin, Priyanka Krishnan, Luis Martinez-Nater, Bharati Mendhe, Sadanand Fulzele, Yutao Liu, Julie A. Mattison, Mark W. Hamrick

**Affiliations:** 1 Medical College of Georgia, Augusta University, CB1116 Laney Walker Blvd, Augusta, GA 30912, USA; akhayrullin@augusta.edu (A.K.); pkrishnan@augusta.edu (P.K.); bmendhe@augusta.edu (B.M.); yutliu@augusta.edu (Y.L.); 2 School of Medicine, Universidad Central Del Caribe, Bayamon, PR 00960, USA; 116lmartinez@uccaribe.edu (L.M.-N.); sfulzele@augusta.edu (S.F.); 3 National Institute on Aging, National Institutes of Health, Bethesda, MD 20892, USA; mattisonj@mail.nih.gov

**Keywords:** aging, neutral sphingomyelinase 2, cell-cell communication, extracellular vesicles

## Abstract

Extracellular vesicles (EVs), including exosomes and microvesicles, function in cell-to-cell communication through delivery of proteins, lipids and microRNAs to target cells via endocytosis and membrane fusion. These vesicles are enriched in ceramide, a sphingolipid associated with the promotion of cell senescence and apoptosis. We investigated the ceramide profile of serum exosomes from young (24–40 yrs.) and older (75–90 yrs.) women and young (6–10 yrs.) and older (25–30 yrs.) rhesus macaques to define the role of circulating ceramides in the aging process. EVs were isolated using size-exclusion chromatography. Proteomic analysis was used to validate known exosome markers from Exocarta and nanoparticle tracking analysis used to characterize particle size and concentration. Specific ceramide species were identified with lipidomic analysis. Results show a significant increase in the average amount of C24:1 ceramide in EVs from older women (15.4 pmol/sample) compared to those from younger women (3.8 pmol/sample). Results were similar in non-human primate serum samples with increased amounts of C24:1 ceramide (9.3 pmol/sample) in older monkeys compared to the younger monkeys (1.8 pmol/sample). In vitro studies showed that primary bone-derived mesenchymal stem cells (BMSCs) readily endocytose serum EVs, and serum EVs loaded with C24:1 ceramide can induce BMSC senescence. Elevated ceramide levels have been associated with poor cardiovascular health and memory impairment in older adults. Our data suggest that circulating EVs carrying C24:1 ceramide may contribute directly to cell non-autonomous aging.

## 1. Introduction

The public health burden of age-related diseases is increasing rapidly as the aging population grows globally. The care provided to those with Alzheimer’s disease (AD) in 2016 reached an estimated economic value of $221.3 billion, and the healthcare cost of osteoporotic fractures in the US was estimated at $14 billion. Emerging patterns of disease progression suggest that degenerative changes in one organ or system are likely to contribute to degenerative changes in other organs and systems. For example, reductions in lean mass and bone loss have both been observed to precede the age-related development of cognitive impairment and AD [1,2]. Thus, cross-talk among various cells, tissues and organs may underlie non-autonomous aging in different cell and tissue populations. This concept is supported by studies in which young cells exposed to aged serum exhibited changes characteristic of older cells [3,4].

A barrier to progress in correcting the problem of age-related tissue dysfunction is the poor understanding of the molecular and cellular mechanisms underlying these non-autonomous cellular communication pathways. Exosomes are small (40–150 nm) and microvesicles are larger (>100 nm) membrane-derived structures that are released into the extracellular space by a variety of cell types [5]. These membrane-bound extracellular vesicles (EVs) can transport proteins, lipids and mRNAs between cells, delivering these molecules to target cells via endocytosis and membrane fusion [5,6]. EV-based cell–cell communication therefore represents a novel pathway for epigenetic reprogramming of target cells [7]. EVs are highly enriched in the sphingolipid ceramide [8,9], which is known to promote cell senescence and apoptosis [10,11,12,13]. In addition, EVs play a key role in a number of pathologies in vivo such as cancer metastasis [14,15] and neurodegenerative disease [9,13,16,17]. Thus, EV-derived ceramide is one potential aging factor that may promote degeneration in multiple organs and tissues.

A number of studies have shown that the liver is a primary source of circulating ceramide [18,19]; ceramide levels in the liver increase with age [20], and ceramide production is elevated with exposure to age-associated stimuli such as reactive oxygen species [21] and inflammatory cytokines [11,22]. Loss of stem cells in musculoskeletal tissues contributes to tissue dysfunction [23,24]; these stem cells endocytose EVs in vitro and in vivo [25], and ceramide induces cell death and senescence in a variety of cell types [10,12]. There are, however, numerous forms of ceramide including short, medium, long, and very long-chain ceramides that differ from one another primarily in their acyl chain lengths. Very long-chain ceramides are, in particular, associated with mitochondrial damage and cell death [26] and are known to increase in the serum of heart failure patients [27]. While ceramides are thought to play a role in aging and cell death, the specific ceramide species involved in tissue dysfunction are not well understood. Here we employ a highly translational approach utilizing EVs isolated from serum samples of young and aged human subjects and non-human primates to define age-related changes in various ceramides. Data from the initial lipidomic analyses are then combined with functional in vitro studies to determine the role of specific ceramides in cellular senescence. Finally, we examine age-related changes in ceramide synthase expression to identify potential therapeutic targets for reducing ceramide production with aging.

## 2. Materials & Methods

### 2.1. Serum Samples, EV Isolation, and EV Characterization

**Figure 1:**
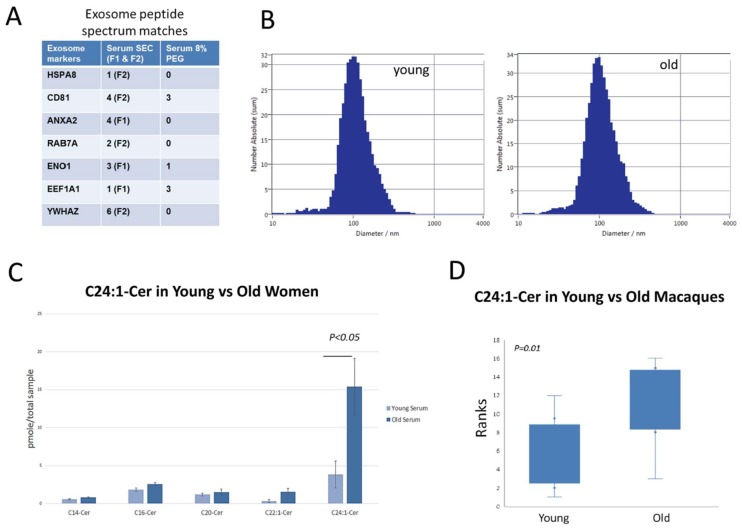
Isolation and characterization of old and young EVs. (**A**) Peptide spectrum matches for a single human serum EV sample isolated using either size-exclusion chromatography (SEC) or 8% polyethylene glycol (PEG). The first two EV fractions (F1/F2) from SEC isolation show a greater number of known EV markers compared to PEG isolation. (**B**) Zetaview quantification of serum EV particle size from young (25–40 yrs) and aged (75–90 yrs) Caucasian, female donors. (**C**) Ceramide lipidomic profile of EVs isolated from serum of young and older women (*n* = 5 per age group, error bars = SD). Note that EVs from older women are highly enriched in C24:1 ceramide. (**D**) Box-and-whisker plots of rank-transformed C24:1 ceramide values of EVs from rhesus monkeys. EVs from the serum of aged monkeys (*n* = 8) show a significant increase in C24:1 ceramide compared to serum EVs isolated from younger monkeys (*n* = 8). Values were converted to ranks and single-factor ANOVA was performed with age as the factor.

We previously used polyethylene-glycol (PEG)-based EV isolation due to the relatively high yields of EVs compared to standard ultracentrifugation approaches [28,29]. Recently it has been shown that ultracentrifugation can alter the structural properties of EVs and that samples isolated using PEG can in some cases contain an overabundance of soluble plasma proteins [30]. Size-exclusion chromatography (SEC) is one approach for EV isolation that retains the structural and biophysical properties of EVs and also excludes the majority of soluble plasma proteins [31,32]. We compared the PEG and SEC isolation procedures using three 1 mL human serum samples (Bioreclamation IVT, Baltimore, MD, USA) that were pooled, mixed and aliquoted into three 1 mL vials. We isolated EVs from one aliquot using 8% PEG, one aliquot using 8% PEG plus SEC, and one aliquot using SEC alone (Izon, Inc., Medford, MA, USA). For the SEC analyses we used the Izon qEV original columns. Following the manufacturer’s procedures, the initial 3 mL void volume was discarded, and the subsequent 2 mL was retained as the F1 (1 mL) and F2 (1 mL) fraction, respectively. The resulting EV populations were analyzed for the top 25 known exosome markers in Exocarta (http://exocarta.org/exosome_markers) using the Orbitrap Fusion Tribrid Mass Spectrometer in the Augusta University Proteomics Core Facility. Representative data files from these analyses are included as supplemental data, and for proteomics the F1 and F2 SEC fractions were analyzed separately. We have submitted all relevant data of our experiments to the EV-TRACK knowledgebase (http://evtrack.org/review.php; EV-TRACK ID: EV180075). Analyses were repeated in duplicate. Results indicate that, consistent with the studies referenced above, SEC yields relatively pure samples demonstrating multiple EV markers (Figure 1A).

We then obtained five serum samples of young women (age 25–40) and five samples of older women (age 75–90), all Caucasian, non-diabetic, non-smokers from ReproCell (Beltsville, MD, USA) for EV isolation using SEC. Females were selected since they show a higher incidence of both Alzheimer’s disease and osteoporosis. Nanoparticle tracking analysis was performed using the ZetaView instrument from Particle Matrix. Serum samples of both young and older women show particle sizes in the 100 nm range, consistent with the known size of exosomes (Figure 1B). We obtained serum samples from eight young (6–10 yrs.) and old (25–30 yrs.) healthy rhesus macaques (*Macaca mulatta*) from the National Institute on Aging and isolated EVs from these samples using SEC as we did for the human samples. 

### 2.2. Lipidomic Analysis of Serum EVs

Serum EV samples isolated from the young and older women described above as well as the samples isolated from young and aged monkeys were analyzed for short-, long-, and very long-chain ceramides at the lipidomic core facility at the Medical University of South Carolina (MUSC), Charleston, SC. Samples were from pooled F1 and F2 fractions isolated using SEC and analyzed on a Thermo Scientific High-Performance Liquid Chromatography-tandem Mass Spectrometry (HPLC-MS/MS) system; data was normalized to levels of inorganic phosphate/sample.

### 2.3. In Vitro Studies of Exosome Uptake

We isolated extracellular vesicles from the serum of adult humans (25–40 yrs.) using SEC as described above for human and monkey serum samples. EVs were stained with the lipophilic membrane dye PKH67 to label extracellular vesicles. We then treated primary human BMSCs (Lonza, MD, USA) with PKH67-labeled EVs. Cells were imaged using confocal microscopy in the Medical College of Georgia Cell Imaging Core Facility.

### 2.4. Loading of C24:1 Ceramide into Exosomes and Analysis of Cell Senescence

C24:1 ceramide (Cayman Chemical, MI) was dissolved in ethanol at a concentration of 1 mg/mL. The solubility of C24:1 ceramide in ethanol is approximately 5.5 mg/mL per manufacturer specifications. We loaded serum EVs isolated from a single mouse with ceramide by adding the EV pellet to the C24:1 ceramide solution, mixing vigorously, incubating at 30 °C for 1 h, purifying the EV pellet again, and then resuspending in de-ionized water (DIW). EVs are loaded effectively with C24:1 ceramide because it is highly hydrophobic. The ceramide-loaded EV suspension was centrifuged at 500× *g* for 5 min followed by 2000× *g* for 30 min at 4 °C to remove particles. The supernatant was removed, PEG solution added (8% PEG final concentration) and the sample incubated at 4 °C overnight. Samples were centrifuged the following day at 12,000 rpm for 1 h at 4 °C. The supernatant was then decanted and the resulting pellet was suspended in 200 μL of PBS. Control (unloaded) EVs were added to the ethanol solution that contained no C24:1 ceramide, sonicated, pelleted, and then resuspended in DIW. Lipidomic analysis on the ceramide-loaded EVs or unloaded (control) EVs was performed to verify effective loading. We then treated primary mouse BMSCs with the unloaded (control) serum EVs or EVs loaded with C24:1 ceramide. As an additional control we processed C24:1 ceramide in solution as we processed EVs. Cells were treated with 50ug/mL of exosomes, 10,000 Mouse BMSCs/well (24 well plate), or with the solution that included C24:1 ceramide but no EVs (Figure included as supplemental data). Primary BMSCs were isolated from femur bone marrow of adult mice 4–6 months age using procedures we have described previously [24,33,34]. Cells were stained for senescence-associated beta-galactosidase (β-gal) as a marker of cell senescence.

### 2.5. Real-Time PCR Analysis of Sphingomyelinase Expression

The liver is the primary source of circulating ceramide [18,19]. Ceramide can be produced in the liver in two ways, either by de novo synthesis or by hydrolysis of sphingomyelin [35]. Ceramide synthase 2 (CerS2) is the primary synthase involved in synthesizing very long-chain C24:1 ceramide through the de novo pathway [36,37], whereas neutral sphingomyelinase 2 (nSMase2) is primarily involved in the production of ceramide by hydrolysis of sphingomyelin [35]. We therefore compared the expression of CerS2 and nSMase2 between the livers of aged (22 mo, *n* = 4) and young (6 mo, *n* = 4) adult female mice to determine which pathway may be involved in the elevated C24:1 ceramide observed with age. Livers were snap frozen in liquid nitrogen after mice were euthanized by CO2 overdose and thoracotomy. mRNA was isolated from livers using RNeasy spin columns (Qiagen) following manufacturer specifications. qRT-PCR was performed using the following primer sequences with the average of GAPDH and 18s RNA expression as the normalization control. CerS2 FWD AAGTGGGAAACGGAGTAGCG, CerS2 REV ACAGGCAGCCATAGTCGTTC, nSMase2 FWD ACACGACCCCTTTCCTAATA, nSMase2 REV GGCGCTTCTCATAGGTGGTG.

### 2.6. Statistical Analysis

Lipidomic data were compared using single-factor ANOVA with age as the factor and Fishers LSD test used for post-hoc comparisons. Pairwise comparisons were also performed on rank-transformed data to reduce the influence of outlying observations. *t*-tests were used for pairwise comparisons of ceramide synthase expression.

## 3. Results

### 3.1. C24:1 Ceramide Is Increased with Age in Extracellular Vesicles from Older Human Subjects and Non-Human Primates

Lipidomic analyses of serum extracellular vesicles indicate that serum EVs from older women are highly enriched in C24:1 ceramide (15.4 pmol/sample) and differ significantly (*p* < 0.05) from younger women (3.8 pmol/sample) in this respect (Figure 1C). Similar to the human studies, EVs isolated from aged monkeys show a significant increase (*p* < 0.01) in C24:1 ceramide: 9.3 pmol/sample in old monkeys versus 1.8 pmol/sample in young monkeys (Figure 1D). Values for the non-human primate serum samples were similar to those of the women studies with increased amounts of C24:1 ceramide in older monkeys. These findings are clinically important since C24:1 ceramide is significantly elevated in the serum of older adults with lower cardiovascular fitness [38], a higher risk of cardiovascular disease [39], and memory impairment [40].

### 3.2. Serum EVS Are Endocytosed by Bone Marrow Stem Cells

**Figure 2:**
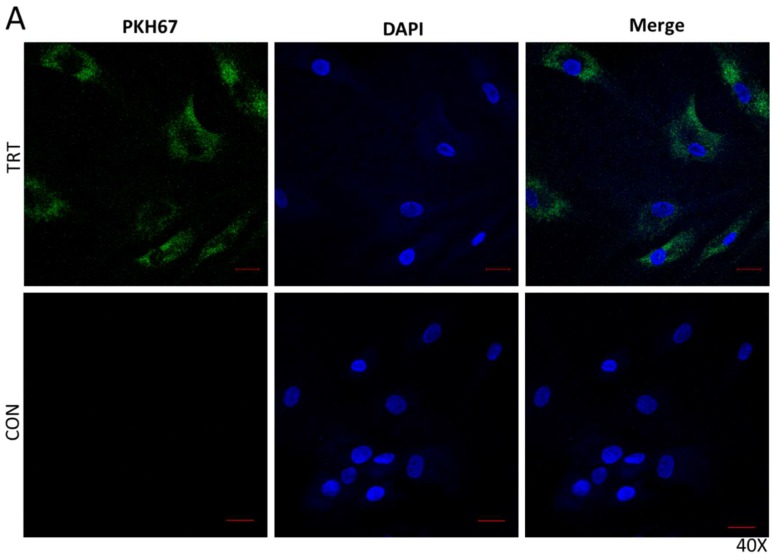

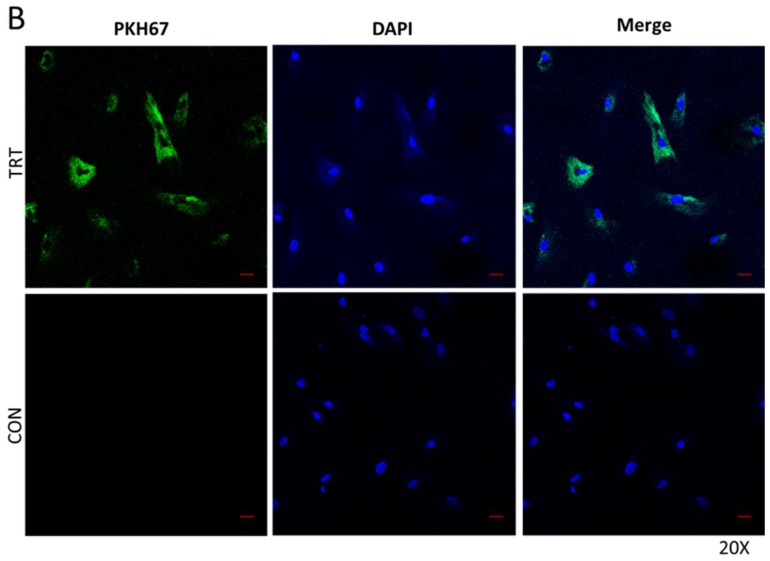
PKH67-labeled serum EVs are endocytosed by human bone marrow mesenchymal stem cells. EVs labeled with PKH67 (green, left and right columns) are endocytosed by cells visualized using confocal microscopy. Cells are shown at high (**A**, 40×) and lower (**B**, 20×) magnification. Images of cells treated with unlabeled (No PKH67 dye) are shown as controls (CON). Blue staining represents nuclear DAPI staining. Scale bars = 20 µm.

Primary human BMSCs were treated with serum EVs isolated from human serum and labeled with PKH67 dye. Clusters of labeled EVs are abundant in the cytosol of bone marrow stem cells, indicating that the cells readily take up these vesicles (Figure 2).

### 3.3. Young EVs Loaded with C24:1 Ceramide Can Induce Cell Senescence

**Figure 3:**
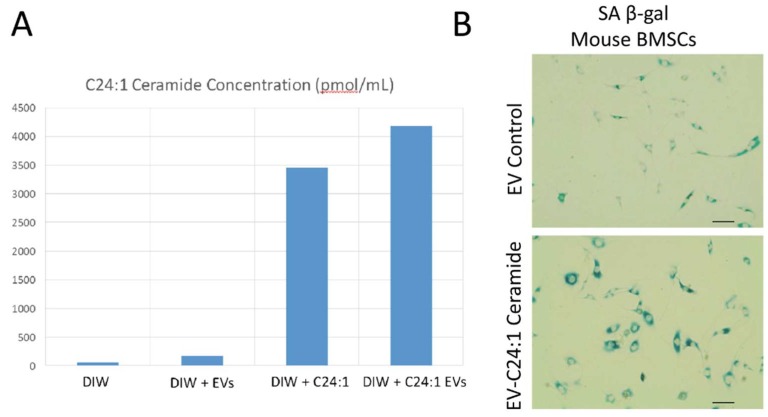
EVs loaded with C24:1 ceramide induce cellular senescence. (**A**) Lipidomic profiles of de-ionized water alone (DIW), DIW + EVs, DIW + C24:1 ceramide, or DIW + EVs loaded with C24:1 ceramide. Bars represent a single sample. (**B**) Staining for β-gal indicated increased senescence in BMSCs treated with C24:1 ceramide-loaded EVs. Scale bars = 40 µm.

The experiments performed above indicate that serum EVs from aged human subjects and non-human primates are enriched in C24:1 ceramide and that primary bone marrow mesenchymal stem cells endocytose serum-derived EVs. We loaded mouse serum EVs with C24:1 ceramide and then treated mouse primary BMSCs with these C24:1-loaded EVs to determine the effects on cellular senescence. Cells were stained for senescence-associated beta-galactosidase (β-gal), a marker of cell senescence. Lipidomic analysis on the ceramide-loaded EVs or unloaded (control) EVs demonstrate that the ceramide-loaded EVs are highly enriched in C24:1 ceramide compared to control, unloaded samples (Figure 3A). Images show strong β-gal staining of BMSCs after treatment with C24:1 ceramide EVs (Figure 3B).

### 3.4. Aging Increases Sphingomyelinase Activity in the Liver

**Figure 4:**
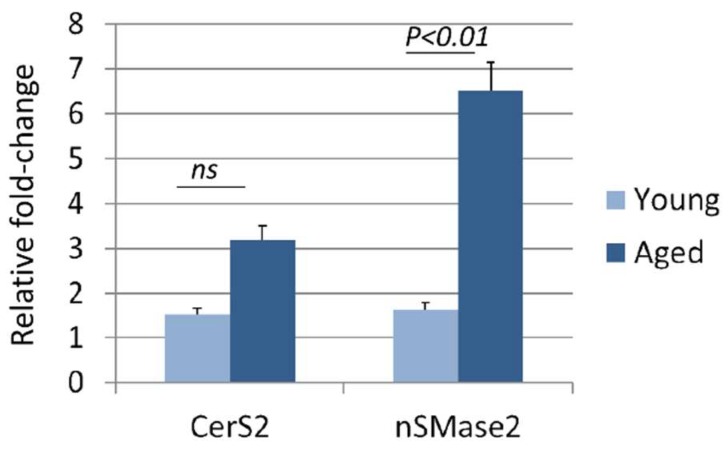
Neutral sphingomyelinase (nSMase2) expression is significantly increased with age in the mouse liver. qPCR results showing the significant increase in nSMase2 expression with age in the mouse liver relative to CerS2 expression (*n* = 4 per age group, error bars represent SEM). nSMase2 produces C24:1 ceramide by hydrolysis of sphingomyelin.

As noted earlier, CerS2 is the primary synthase involved in synthesizing very long chain C24:1 ceramide through the de novo pathway whereas nSMase2 is primarily involved in the production of ceramide by hydrolysis of sphingomyelin. We therefore compared the expression of CerS2 and nSMase2 between livers of aged (22 mo) and young (6 mo) adult female mice to determine which pathway may be involved in the elevated C24:1 ceramide observed with age. Results indicate that CerS2 expression was not significantly altered with age, where nSMase2 was significantly increased with age (Figure 4).

## 4. Discussion

EV-derived transport of lipids, proteins, and small non-coding RNAs is increasingly gaining attention as a mechanism of disease pathogenesis [7]. For example, EVs are now recognized to play key roles in a number of pathologies in vivo such as cancer metastasis [14,15] and neurodegenerative disease [9,13,16,17]. EVs are highly enriched in the sphingolipid ceramide [8,9], which can itself promote cell senescence and apoptosis [10,11,12,13]. These findings are clinically important since elevated levels of circulating ceramide species have recently been linked to the development and progression of Alzheimer’s disease [38] as well as with frailty and declining gait performance with aging [39]. Thus, EV-derived ceramide is a novel cell non-autonomous aging factor that is likely to promote degeneration in multiple organs and tissues. Our work is novel in that the role of EVs in non-autonomous cell aging is relatively unexplored. In addition, while ceramide is known to be involved in cell autonomous aging and senescence [10], its role in tissue crosstalk with aging has not been well-characterized. Moreover, lipid abnormalities are associated with a number of different conditions including metabolic disorders, cardiovascular disease, and neurodegeneration. Lipidomic technologies have the potential to shed new light on disease biomarkers [40], and our data provide evidence that lipidomics can also be applied to the study of extracellular vesicles within the context of aging.

Results presented here indicate that EVs from aged individuals are highly enriched in very long chain C24:1 ceramide. The concentration of circulating extracellular vesicles appears to actually decline with age [41], suggesting that increases in EV-derived ceramide are likely unrelated to an age-associated increase in EV biogenesis. C24:1 ceramide is also significantly elevated in the serum of older adults with lower cardiovascular fitness [42], a higher risk of cardiovascular disease [43], and memory impairment [44]. Additional studies indicate that C24:1 ceramide is important for regulating cell senescence [45] and inducing apoptosis [9]. Ceramides can also suppress anabolic activities in various organs and tissues by blocking Akt and inhibiting mTor signaling [46]. Finally, ceramide has also been found to downregulate amino acid transporters [47,48]. This represents another mechanism by which age-related ceramide accumulation might contribute to cell non-autonomous aging—that is, by essentially “starving” cells to death. All of these studies point to a highly detrimental effect of ceramide accumulation on cell anabolism and cell survival. This point is underscored by a recent study demonstrating that the adipokine adiponectin promotes cellular protection and cell survival by stimulating ceramide efflux from cells via extracellular vesicles [49].

CerS2 is the primary synthase involved in synthesizing very long chain C24:1 ceramide through the de novo pathway [37], whereas nSMase2 is primarily involved in the production of ceramide by hydrolysis of sphingomyelin [35]. Our data showing that nSMase2 expression is increased with age in the mouse liver suggest that elevated C24:1 ceramide in circulating EVs with age is due at least in part to increased hydrolysis of sphingomyelin by nSMase2. Previous studies indicate that reactive oxygen species [21] and inflammatory cytokines [11,22] can induce nSMase2 expression and long-chain ceramide production. ROS, TNFα, and IL-6 also increase with age [50,51,52], and aging is also associated with the elevated expression of nSMase2 [53]. There are a number of potential pharmacological approaches for inhibiting ceramide synthesis with exposure to ROS or inflammatory cytokines, such as blocking nSMase2 activity directly with cambinol or its analogues [54]. These observations suggest that very long-chain ceramides, as well as perhaps shorter length ceramides, may represent potential therapeutic targets for the treatment and prevention of various age-associated conditions.

## Supplementary Materials

Supplementary Materials are available online.
