# Supplementary material for: Very Long-Chain C24:1 Ceramide Is Increased in Serum Extracellular Vesicles with Aging and Can Induce Senescence in Bone-Derived Mesenchymal Stem Cells
Source: Cells. 2019 Jan 10;8(1):37. doi: 10.3390/cells8010037 (PMC6356348; doi:10.3390/cells8010037)

ß-gal assay performed on BMSCs following treatment with vehicle solution or C24:1 ceramide solution processed as solution with loaded exosomes (centrifugation, precipitation, followed by centrifugation)

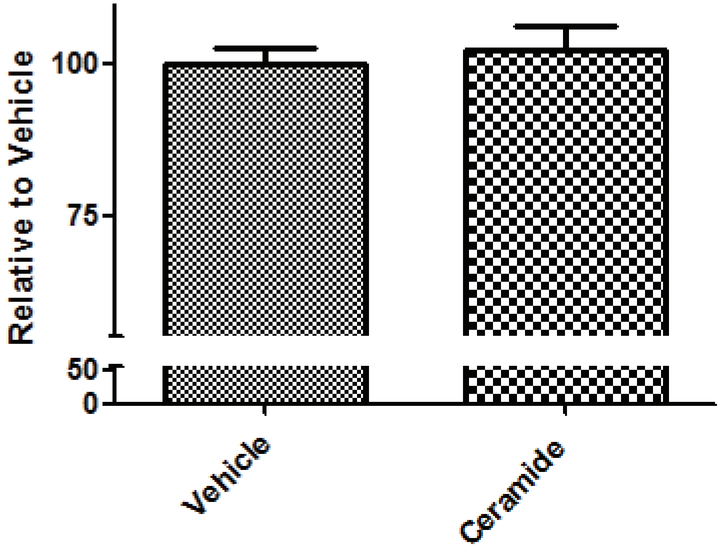

Supplement: Supplementary file 1 [file cells-08-00037-s001.zip › SupplementalData.pdf]
